# Psychological Care Is Mission Critical in ACHD

**DOI:** 10.1016/j.jaccas.2026.107629

**Published:** 2026-04-22

**Authors:** Tabitha G. Moe

**Affiliations:** Honor Health Heart Care, Phoenix, Arizona, USA

**Keywords:** congenital heart disease, postsurgical, psychotherapy, treatment

**Figure:**
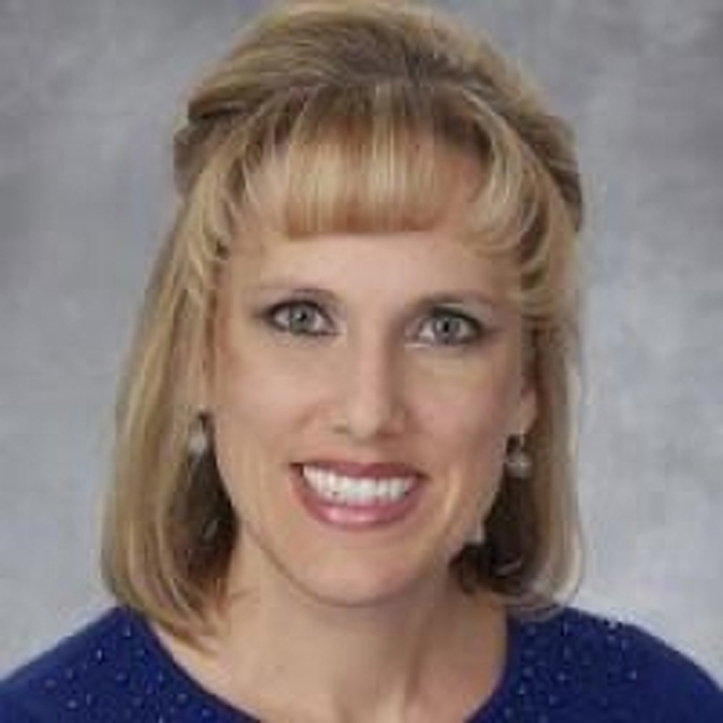

Congenital heart disease (CHD) is no longer solely a pediatric condition. Advances in medical and surgical management have enabled most individuals born with CHD to survive into adulthood, creating a growing population with unique medical and psychosocial needs.^1^ The chronic nature of CHD, coupled with its potential for lifelong complications, underscores the necessity for integrated psychological care as a core component of adult CHD (ACHD) management. In this issue of *JACC: Case Reports*, Finn and Veldtman^2^ describe an adult in transition facing yet another surgical procedure.

Individuals with ACHD often face a range of psychological challenges. These may include anxiety related to health uncertainty, depression stemming from physical limitations, and social isolation due to perceived differences from peers.^3^ The transition from pediatric to adult care can also be fraught with feelings of abandonment or loss, further complicating psychological adjustment. Research consistently demonstrates higher rates of mood and anxiety disorders in the ACHD population compared with the general adult population.^4^

Integrated psychological care involves the seamless incorporation of mental health services into routine cardiology follow-up and disease management. This approach recognizes the bidirectional relationship between psychological well-being and physical health.^5^ Finn and Veldtman^2^ describe a specific course of preoperative interventions to optimize resilience and mental health in preparation for a planned surgical procedure.

Depression and anxiety can negatively affect self-management behaviors, medication adherence, and even physiological outcomes such as arrhythmia frequency and blood pressure control. Conversely, effective psychological support can promote resilience, improve quality of life, and optimize clinical outcomes.^1^

## Key Components of Integrated Care

• Routine screening: Regular screening for depression, anxiety, and posttraumatic stress should be standard practice in ACHD clinics.^4^
• Multidisciplinary collaboration: Care teams should include psychologists, social workers, and psychiatric professionals alongside cardiologists and nurses.
• Tailored interventions: Cognitive-behavioral therapy, mindfulness-based interventions, and peer support groups have shown promise in addressing the specific needs of this population.^3^
• Family and social support: Given the lifelong nature of CHD, family dynamics and social support systems are crucial and should be integrated into care plans.
• Transition support: Structured programs to facilitate the transition from pediatric to adult services can reduce psychological distress and improve continuity of care.^1^

Despite the clear benefits, several barriers impede the widespread adoption of integrated psychological care. These include limited resources, lack of trained mental health professionals within cardiology settings, and stigma surrounding mental health issues. Overcoming these challenges requires institutional commitment, targeted training, and advocacy for policy changes that recognize psychological care as an essential part of management.^3^ Finn and Veldtman^2^ describe a specific early intervention before a planned upcoming procedure to proactively manage symptoms and improve psychological outcomes.

The integrated psychological care for ACHD is not a luxury, but a necessity. As survival improves and the ACHD population expands, the medical community must embrace a holistic approach that addresses both physical and psychological health.^1^ Future research and policy should focus on developing sustainable models of integrated care and evaluating their impact on long-term outcomes. Only then can we truly meet the needs of this complex and resilient patient population.

## Funding Support and Author Disclosures

The author has reported that she has no relationships relevant to the contents of this paper to disclose.

## References

[1] Moons P., Luyckx K., Thomet C. (2021). Patient-reported outcomes in adults with congenital heart disease: interdisciplinary care and research. Eur Heart J.

[2] Finn M.T.M, Veldtman G.R (2026). Integrated psychological care of an adult with congenital heart disease. JACC Case Rep.

[3] Latal B. (2016). Psychological adjustment and quality of life in children and adults with congenital heart disease. World J Cardiol.

[4] Kovacs A.H., Saidi A.S., Kuhl E.A. (2009). Depression and anxiety in adult congenital heart disease: predictors and prognosis. Int J Cardiol.

[5] Hövels-Gürich H.H. (2016). Psychological adjustment in adults with congenital heart disease. World J Pediatr Congenit Heart Surg.

